# Duodenal GIST: a single center experience

**DOI:** 10.1007/s00384-012-1432-8

**Published:** 2012-02-22

**Authors:** Alexander Beham, Inga-Marie Schaefer, Silke Cameron, Katharina von Hammerstein, Laszlo Füzesi, Giuliano Ramadori, Michael B. Ghadimi

**Affiliations:** 1 Department of General and Visceral Surgery, University Medical Center Göttingen, Göttingen, Germany; 2 Department of Pathology, University Medical Center Göttingen, Robert-Koch-Straße 40, 37075 Göttingen, Germany; 3 Department of Gastroenterology and Endocrinology, University Medical Center Göttingen, Göttingen, Germany

**Keywords:** GIST, Duodenum, *KIT* mutation, *PDGFRA* mutation, Comparative genomic hybridization

## Abstract

**Purpose:**

The duodenum as primary site for gastrointestinal stromal tumors (GISTs) is rare and mitotic rate, tumor size, type of mutation and number of chromosomal aberrations have prognostic implications.

**Methods:**

We analyzed the outcome of 13 patients with duodenal GISTs who underwent surgical tumor resection. Either segmental duodenectomy or pylorus-preserving duodenopancreatectomy was performed. The tumors were histopathologically examined and the risk of progression was assessed based on tumor size and mitotic count. Additionally, mutation analysis of the *KIT* and *PDGFRA* receptor tyrosine kinase genes and comparative genomic hybridization (CGH) were performed in all cases.

**Results:**

Eight patients underwent segmental duodenectomy and five patients were treated with pylorus-preserving duodenopancreatectomy. None of the five GISTs with low or no risk for malignancy according to the Miettinen classification developed tumor progress. In contrast, five of eight cases (62.5%) with high-risk tumors revealed tumor progress, and four of these patients died (50%). The median overall survival for all patients was 66 months, and the median disease-free survival 41 months. The operative procedure and type of mutation did not correlate with long-term survival. CGH analysis displayed −15q in 12/13 tumors, and −1p in 11/13 cases as characteristic chromosomal aberrations for intestinal origin. Notably, −22q was present in three of four cases with tumor progress.

**Conclusions:**

Both segmental duodenectomy and pylorus-preserving duodenopancreatectomy are appropriate options to treat duodenal GIST and should be implemented depending on resectability and the patient's performing state. The Miettinen classification and CGH findings correlate with the clinical course.

## Introduction

Gastrointestinal stromal tumors (GISTs) are supposed to arise from the interstitial cells of Cajal or their precursors, located throughout the muscular wall of the gastrointestinal tract. They occur at an incidence of 10–20/million per year and at a median age of 55–60 years [1–4]. They arise mostly in the stomach (60%), followed by the small intestine (35%) and rectum, esophagus, omentum, and mesentery (<5%) [2]. Duodenal GISTs account for only <5% but make up 30% of primary duodenal tumors [5]. Most cases occur sporadically, but 5% occur in the context of a familial syndrome (i.e., neurofibromatosis type 1, Carney triad) [2]. They usually present with abdominal pain to due obstruction, anemia, or gastrointestinal bleeding from a central ulceration. Small duodenal GISTs may be incidental findings during gastroscopy. Grossly, GISTs typically present as a sharply demarcated mass lesion without lymphatic spread, arising in the submucosa [6]. Histologically, spindle cell (70%), epithelioid (20%) or mixed type differentiation can be observed, depending on tumor site [3]. Tumor size, mitotic activity and anatomic site are currently used to predict malignant courses according to the modified Miettinen classification [4]. Furthermore, the results of mutation analysis of the *KIT* and *PDGFRA* gene and comparative genomic hybridization (CGH) are employed as additional prognostic factors with impact on diagnosis and therapy [2, 7]. The individualized application of tyrosine kinase inhibitors in patients with high-risk GISTs, certain cases of intermediate-risk GISTs, and/or incomplete surgical resection has been established during the past years. However, the role of surgical treatment remains important since only complete resection of primary GISTs is curative [1, 5, 6, 8]. The optimal surgical technique for duodenal GISTs remains to be determined [1, 5, 6, 8–13]. Therefore, a comprehensive risk assessment with regard of patient outcome is necessary to compare the beneficial effects of limited or major surgery for duodenal GISTs. The purpose of this study was to compare the efficacy of duodenal segmentectomy and pylorus-preserving duodenopancreatectomy, and (neo-) adjuvant therapy in 13 primary duodenal GISTs with regard of recurrence rate and survival.

## Materials and methods

### Patients and tumor specimens

**Fig. 1:**
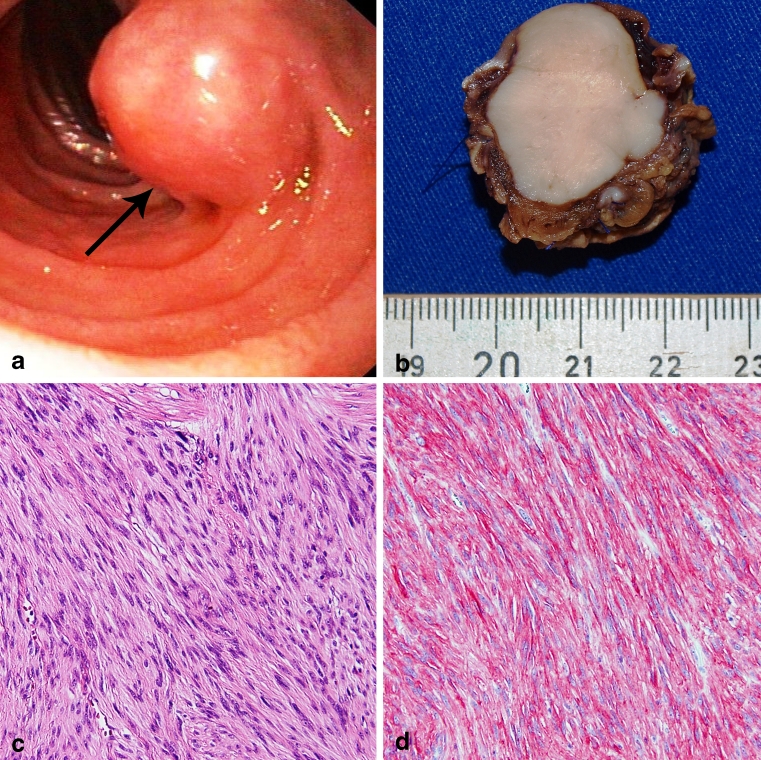
Morphologic findings in a case of duodenal GIST (patient 12): endoscopy revealed a submucosal mass lesion in the duodenum covered by normal duodenal mucosa (**a** *arrow*). Grossly, the tumor measured up to 2.5 cm in size with sharply defined margins and a pale-white, solid cut surface (**b**). Histologically, the tumor was of spindle cell differentiation without cellular atypia (**c** hematoxylin–eosin stain) and showed marked expression of CD117 (cKIT, **d**) (×100)

Thirteen patients including 7 men and 6 women with a mean age of 69.4 years (range, 58–75 years), who underwent surgical resection of duodenal GISTs, were included in this study. Formalin-fixed and paraffin-embedded tumor samples were examined. Immunohistochemical staining with CD117 (cKIT; Dako, Glostrup, Denmark), PDGFRA (Neo Markers, Fremont CA, USA), CD34 (Neo Markers), smooth-muscle actin (Zytomed Systems, Berlin, Germany), desmin (Invitrogen, Berlin, Germany), S-100 (Neo Markers), and Ki67 (Zytomed Systems) was performed in all cases (Fig. 1). Assessment of maximal tumor size, histologic growth pattern, and mitotic count in 50 high power fields (HPFs) was performed independently of clinical variables. The malignant potential was estimated based on tumor size, mitotic count, and location according to the updated AFIP criteria published in 2006 by Miettinen and Lasota [4]. Survival data could be obtained for all patients by reviewing the clinical records and direct communication with the attending physicians. The surgical procedure, including segmental duodenectomy or pylorus-preserving partial duodenopancreatectomy according to Traverso-Longmire was registered. Based on tumor size and site, the decision between both operative procedures was made intraoperatively. Additionally, preoperative and postoperative therapy with tyrosine kinase inhibitors was assessed. The ethical committee of the University Medical Center Göttingen, Germany, approved of the experiments performed in this study (No. 26/12/10).

### Mutation analysis

Mutation analysis of *KIT* exons 9, 11, 13, and 17, as well as *PDGFRA* exons 12, 14, and 18, was performed using direct sequencing of PCR products as described previously [14].

### Comparative genomic hybridization

CGH from formalin-fixed and paraffin-embedded tumor tissue specimens was performed essentially as described previously [7].

### Statistical analysis

All statistical analysis was performed by using the software program Statistica 9.1 (StatSoft, Hamburg, Germany). Disease-free survival and overall-survival were estimated by the Kaplan–Meier method. The groups were compared with the non-parametric log–rank test.

## Results

### Follow-up

**Fig. 2:**
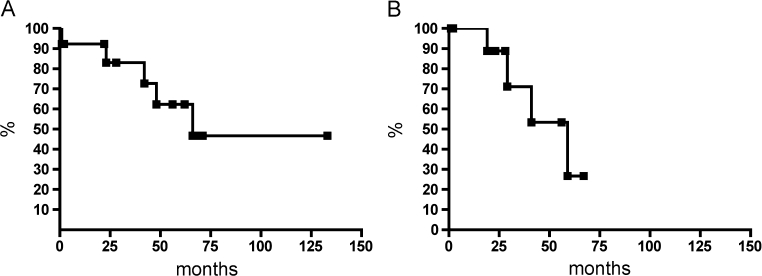
Kaplan–Meier estimator demonstrates overall (**a**) and disease-free survival (**b**) for the 13 patients with duodenal GIST

Survival data could be obtained for all patients. None of the five GISTs with low or no risk for malignancy according to Miettinen's criteria developed tumor progress. In contrary, five of the eight cases with high risk of malignancy revealed tumor progress (62.5%) and four of these patients died (50%). Overall survival and disease-free survival are demonstrated in Fig. 2 with a median overall survival of 66 months and a median disease-free survival of 41 months.

**Fig. 3:**
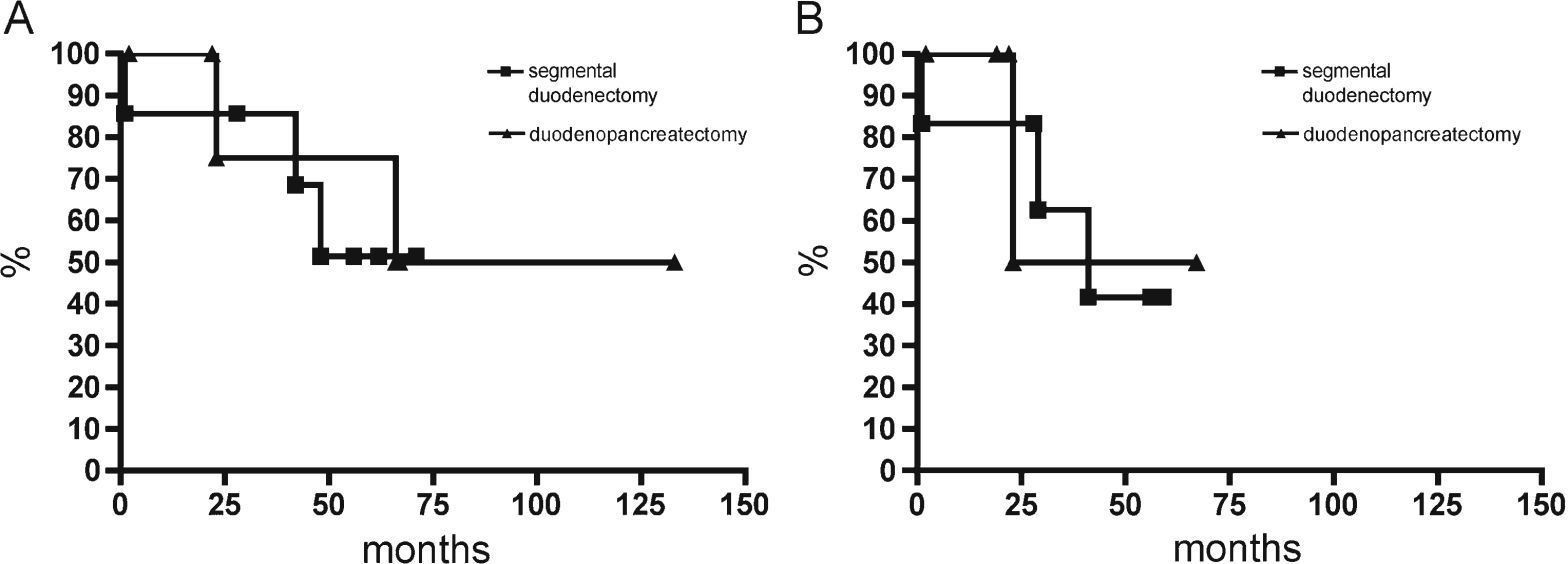
Kaplan–Meier estimator shows the overall survival (**a**) and disease-free survival (**b**) for the 13 patients with duodenal GIST comparing segmental duodenectomy vs. duodenopancreatectomy

Surgical procedures included segmental duodenectomy in eight patients, one of which was treated with secondary pylorus-preserving duodenopancreatectomy due to anastomotic leakage and primary pylorus-preserving duodenopancreatectomy according to Traverso-Longmire in five patients. There was no significant survival benefit for one or the other surgical approach regarding overall survival (*P* = 0.6993) or recurrence-free survival (*P* = 0.8629) (Fig. 3). One of the patients with segmental duodenectomy died within the first 30 days due to cardiac arrhythmia. In contrast, none of the patients with duodenopancreatectomy died. The postoperative complications are listed in Table 1.

**Table 1:** The perioperative complications of duodenal segmentectomy and pylorus-preserving duodenopancreatectomy (Traverso-Longmire) are demonstrated

Operative procedure	Perioperative complications (patient)
Duodenal segmentectomy	Pulmonary edema (1)
	Hypoxic encephalopathy from unknown respiratory failure (1)
	Cardiac arrhythmia (8, 10)
	Anastomotic leakage, secondary Traverso-Longmire after 13 days (11)
	Insulin-dependent diabetes mellitus (11)
	Stenosis of biliodigestive anastomosis (11)
Traverso-Longmire	Insulin-dependent diabetes mellitus (2)
	Impaired healing, wound revision; infected bilioma (5)
	Pancreas fistula (9, 13), impaired healing (9)

One patient (patient 6) presented with a synchronous gastric GIST of 1.5 cm size and spindle cell differentiation. The presence of a *KIT* exon 11 mutation (c.1648_1662del; p.K550_E554del) and chromosomal losses at 14q and 22q suggested a synchronous primary gastric GIST rather than a metastasis. Both tumors were resected and the patient received imatinib for 3 years. After 71 months, the patient had no evidence for relapse. One patient (patient 5) had a synchronous liver metastasis, which was treated three times by radiofrequency ablation and hemihepatectomy after 53 months. The primary reason for the operative intervention and resection of the GIST in this case was the impairment of food passage. At this time (2002), neoadjuvant therapy was not yet considered. Subsequently, the patient was treated with imatinib (400 mg/d) for 4 years followed by sunitinib (50 mg/d) and survived 69 months. Three patients [2, 4, 7] developed liver metastases at 19, 29, and 59 months after resection of the primary tumor, respectively. Patient 2 is still alive at 133 months, patient 4 died after 48 months, and the patient 7 is still alive at 62 months. There was, however, no significant difference in the overall survival for patients with and without synchronous liver metastases (*P* = 0.6184). Two patients [1, 2] had local tumor recurrence after 41 and 19 months, the former of which survived 42 months, and the latter was operated for local recurrence (R2), received subsequently chemotherapy with adriamycin/ifosfamide (four cycles) (see Table 2) and is still alive at 133 months under imatinib therapy (400 mg/day). The overall survival in the seven patients without any evidence of tumor recurrence was not statistically different from the patients with evidence for remaining/recurrent GIST (*P* = 0.4816). Two patients [11, 13] received preoperative (i.e., neoadjuvant) treatment with imatinib. Patient 11 was treated with 400 mg/day imatinib for 6 months, and patient 13 (with an exon 9 mutated GIST) received 800 mg/day for 3 months in order to reduce tumor size and allow complete surgical resection (Table 2).

**Table 2:** Clinopathological data of the 13 patients with primary duodenal gastrointestinal stromal tumors

Patient	Age	Sex	Tumor size (cm)	Histologic type	Mitotic count/50 HPF	Risk of progression (Miettinen)	Operative procedure	Preoperative therapy	Postoperative therapy	Other malignancis	Site of recurrence/metastasis
1	74	W	10	Spindle cell	6	High	Duodenal segmentectomy	–	–	–	Local
2	62	M	10	Spindle cell	30	High	Traverso-Longmire	–	4 cycles adriamycin/ifosfamide, imatinib 400 mg/day for 6 months, 200 mg/day for 3 years, 400 mg/day for 6 years up to date	–	Local, HEP
3	58	M	15	Spindle cell	10	High	Duodenal segmentectomy	–	–	–	–
4	70	M	12.5	Spindle cell	50	High	Duodenal segmentectomy	–	Imatinib 400 mg/day for 6 months	–	HEP
5	62	W	8	Spindle cell	100	High	Traverso-Longmire	–	Imatinib 400 mg/day for 4 years; sunitinib 50 mg/day for 1 month	–	HEP^a^
6	71	M	1.8	Mixed spindle cell/epithelioid	2	No risk	Duodenal segmentectomy	–	Imatinib 400 mg/day for 3 years	Prostatic adeno-carcinoma	Gastric GIST^a^
7	74	W	9	Spindle cell	7	High	Traverso-Longmire	–	–	–	HEP
8	67	M	3.7	Spindle cell	0	Low	Duodenal segmentectomy	–	–	HNPCC-syndrome, colonic adeno-carcinoma	–
9	75	W	4	Spindle cell	1	Low	Traverso-Longmire	–	–	Urinary bladder transitional cell carcinoma	–
10	75	W	10	Spindle cell	1	High	Duodenal segmentectomy, atypical gastrectomy due to perforation during gastroscopy	–	–	–	–
11	68	M	4.5	Spindle cell	0	Low	Duodenal segmentectomy with cholecystectomy, secondary Traverso-Longmire	Imatinib 400 mg/day for 6 months	–	–	–
12	75	W	2.5	Spindle cell	2	Low	Duodenal segmentectomy	–	–	–	–
13	71	M	5.2	Spindle cell	1	High	Traverso-Longmire	Imatinib 800 mg/day for 3 months	Imatinib 800 mg/day planned for 1 year	–	–

*HPF* high power fields, *HEP* liver metastasis

^a^Synchronous

### Clinicopathological characteristics

**Fig. 4:**
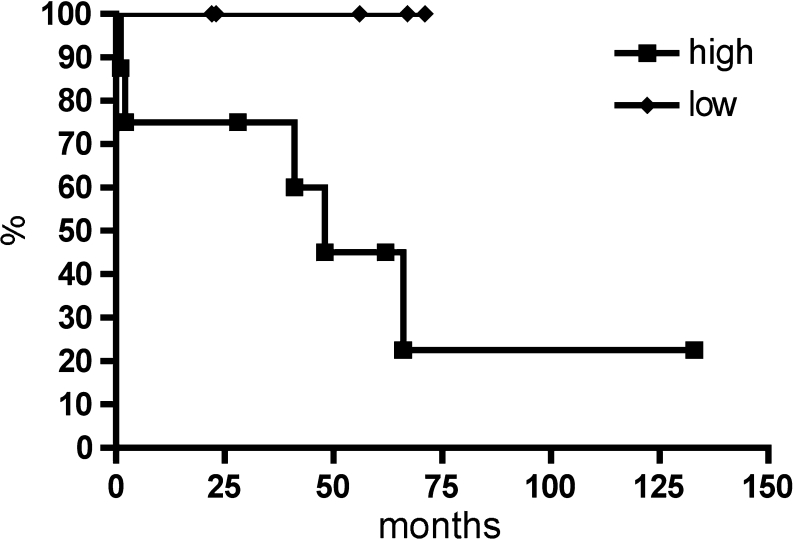
Kaplan–Meier estimator demonstrates overall survival for the 13 patients with duodenal GIST comparing low-risk vs. high-risk duodenal GIST

Table 2 summarizes the clinicopathological characteristics for 13 patients with duodenal GISTs. The mean age was 69.4 years, and there was no significant correlation between age (≤71 vs. >71 years; *P* = 0.9139) and tumor recurrence. The mean tumor size was 7.4 cm (range 1.8–15 cm). There was no significant correlation between tumor size and tumor recurrence (≤10 vs. >10 cm; *P* = 0.2141). Grossly, the tumors were sharply demarcated without infiltrative growth (Fig. 1). Histologically, 12 tumors were of spindle cell differentiation, and one tumor was of mixed type differentiation combining spindle cell and epithelioid areas. There was no significant correlation between the histologic subtype (spindle cell vs. mixed type) and tumor recurrence (*P* = 0.3618). Immunohistochemically, all tumors expressed CD117, four tumors PDGFRA, ten tumors CD34, eight tumors smooth-muscle actin, one tumor desmin, and S-100 was positive only in the intermixed dendritic cells. The mitotic rate per 50 HPFs ranged from 0 to 100 (mean 16/50 HPFs). The risk of malignant behavior according to Miettinen resulted in one case with no risk, four cases of low risk and the remaining eight cases with high-risk potential [4]. There was no significant association of mitotic rate and recurrence rate (≤5 vs. >5 mitoses/50 HPFs; *P* = 0.8264). There was a tendency towards differences in the overall survival for patients with high-risk potential compared to patients with low-risk potential, however, due to the small patient number the findings, were not statistically significant (*P* = 0.0556) (Fig. 4).

### Mutation analysis

**Fig. 5:**
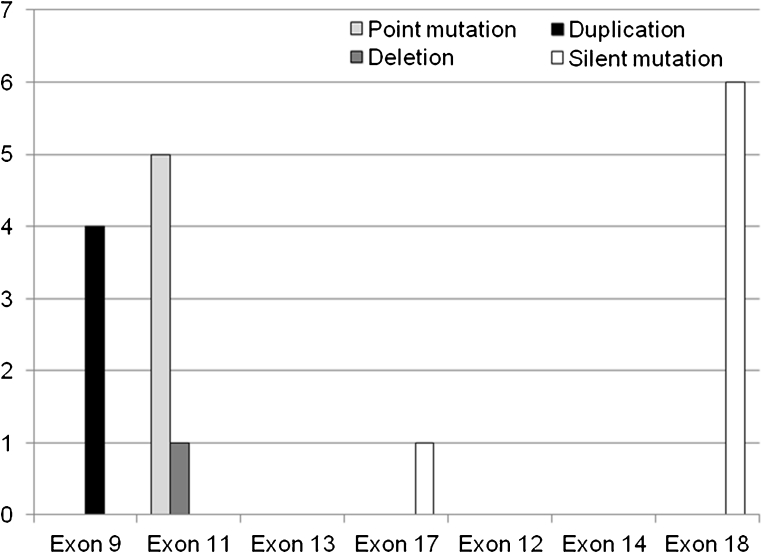
Results of *KIT* exon 9, 11, 13, and 17 and *PDGFRA* exon 12, 14, and 18 mutation analyses in 13 duodenal gastrointestinal stromal tumors

Mutation analysis of the *KIT* and *PDGFRA* gene was performed in all cases (Table 3). The total number of mutations per each examined exon for all patients is summarized in Fig. 5. The number of mutations ranged between 0 and 2 mutations per tumor. Three tumors revealed no mutations (so-called “wild-type” GISTs), and ten tumors displayed one mutation. The polymorphism V824V was present in six cases, and a silent *KIT* exon 17 mutation in one case. *KIT* exon 9 harbored the A502_Y503 duplication in all four cases, *KIT* exon 11 displayed the point mutations W557G, V559D, V560E, and the deletion W557_K558. *KIT* exon 13 and *PDGFRA* exon 12 and 14 revealed no mutations.

**Table 3:** Results of comparative genomic hybridization (CGH) and mutational analysis of *KIT* exon 9, 11, 13, and 17 and *PDGFRA* exon 12, 14, and 18 in 13 patients with primary duodenal gastrointestinal stromal tumors

Patient	Clonal net changes	CGH net changes	Gains	Losses	Amplifi-cations	KIT Exon 9	KIT Exon 11	KIT Exon 13	KIT Exon 17	PDGFRA Exon 12	PDGFRA Exon 14	PDGFRA Exon 18
1	−1p, −2p, −11p, −11q, −13q, −15q	6	0	6	0	WT	c.1669 T > G; p.W557G	WT	WT	WT	WT	WT
2	+4p, +4q, +7q, −1p, −15q, −22q, ++5p	7	3	3	1	WT	c.1676 T > A; p.V559D	WT	WT	WT	WT	c.2472 C > T; p.V824V
3	+8q, +9q, −1p, −15q, −22q	5	2	3	0	WT	c.1679 T > A, 1,680 T > G; p.V560E	WT	WT	WT	WT	c.2472 C > T; p.V824V
4	+17p, −1p, −6q, −15q	4	1	3	0	c.1504_1509dup; p.A502_Y503dup	WT	WT	WT	WT	WT	WT
5	+5p, +5q, −1p, −5q, −9p, −9q, −14q, −15q, −18p, −18q, −21q, −22q, ++5q	13	2	10	1	WT	c.1669_1674del; p.W557_K558del	WT	WT	WT	WT	WT
6	No imbalances	0	0	0	0	WT	WT	WT	WT	WT	WT	c.2472 C > T; p.V824V
7	+2q, +7p, +7q, −1p, −13q, −14q, −15q, −18p, −18q, −22q	10	3	7	0	c.1504_1509dup; p.A502_Y503dup	WT	WT	c.2394 C > T; p.I798I	WT	WT	WT
8	−15q	1	0	1	0	WT	WT	WT	WT	WT	WT	WT
9	−1p, −15q	2	0	2	0	WT	c.1676 T > A; p.V559D	WT	WT	WT	WT	c.2472 C > T; p.V824V
10	−1p, −12p, −14q, −15q	4	0	4	0	WT	WT	WT	WT	WT	WT	WT
11	−1p, −2q, −15q	3	0	3	0	c.1504_1509dup; p.A502_Y503dup	WT	WT	WT	WT	WT	c.2472 C > T; p.V824V
12	+5q, −1p, −2p, −2q, −3p, −3q, −11p, −11q, −12p, −15q	10	1	9	0	WT	c.1676 T > A; p.V559D	WT	WT	WT	WT	c.2472 C > T; p.V824V
13	−1p, −2p, −13q, −15q, −22q	5	0	5	0	c.1504_1509dup; p.A502_Y503dup	WT	WT	WT	WT	WT	WT

*WT* wild-type

### Comparative genomic hybridization

**Fig. 6:**
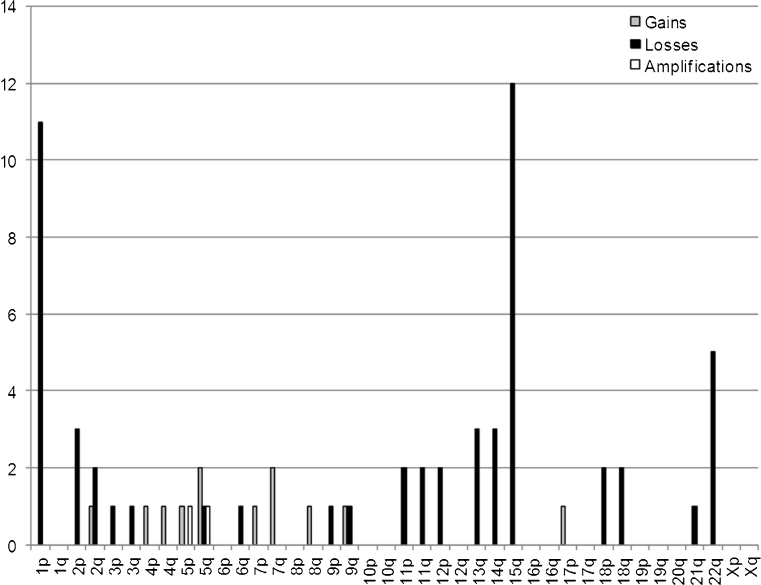
Chromosomal imbalances in 13 duodenal gastrointestinal stromal tumors as detected by comparative genomic hybridization are shown as *bright gray* (gains), *black* (losses), and *dark gray* bars (amplifications) for each chromosome. Losses at 1p, 15q, and 22q are among the most frequently observed aberrations

**Fig. 7:**
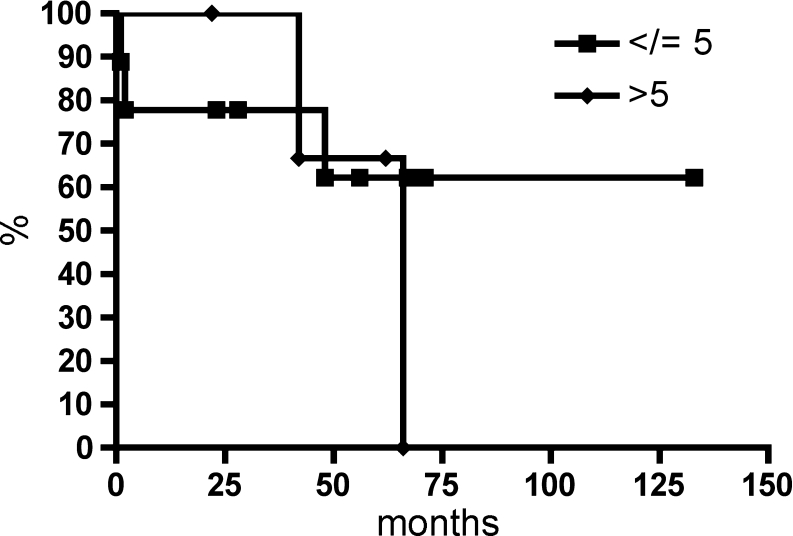
Kaplan–Meier estimator demonstrates overall survival for the 13 patients with duodenal GIST comparing the cases with ≤5 and >5 chromosomal losses as detected by comparative genomic hybridization

CGH was performed in all cases (Table 3). The chromosomal gains and losses of all patients are summarized in Fig. 6. The mean number of aberrations was 5.4 (range 0–13) including a mean of 1 gain (range, 0–3), 4.3 losses (range, 0–10), and 0.16 amplifications (range, 0–1). Patient 6 displayed no chromosomal imbalances. The most frequently observed aberrations comprised losses at 1p (11/13 cases, 84.6%), losses at 15q (12/13 cases, 92.3%), and losses at 22q (5/13 cases, 38.4%). Less than or five losses were not associated with a better survival rate as compared to >5 losses (*P* = 0.0799) (Fig. 7).

## Discussion

Only 4–5% of all GISTs are located in the duodenum [4, 6, 13]. As reported, abdominal pain, anemia, and gastrointestinal bleeding are usually the most common symptoms [13]. Due to the absence of lymph node metastases or infiltrative growth of GISTs, local excision is usually sufficient, but when the tumor is located close to important anatomical structures, pylorus-preserving duodenopancreatectomy may become necessary [6].

We reviewed the outcome of 13 patients, who underwent surgery for duodenal GIST by segmental duodenectomy and pylorus-preserving duodenopancreatectomy.

Based on the criteria proposed by Miettinen et al. [4] for the estimation of the risk of progression, the tumors in the present study were classified as no risk in one case (7.7%), low risk in four cases (30.8%), and high risk in nine cases (69.2%). Six tumors displayed more than five mitoses/50 HPF, thus automatically qualifying as high-risk tumors. Of the seven patients with ≤5 mitoses/50 HPF, two were classified as high risk due to the large tumor size of >5 cm. The mean tumor size of 7.4 cm in the present study is in accordance with previous findings in 90 primary and metastatic duodenal GISTs in which the mean tumor size was 6 cm (range 1.5–31 cm) [13]. The reported prevalence of high-risk cases ranges from 23 to 44% in GISTs of all locations [15–17]. The large number of high-risk tumors in the present study may be explained by both, the location, as no intermediate stage is defined for duodenal GISTs and because of the high mitotic rate in some of our cases. As expected, only the high-risk cases developed recurrence (5/8 cases) as compared to none of the patients with low-risk GIST (0/5). In the present study, 38.5% of the patients developed localized or distant tumor progress. These findings are similar to previous findings in duodenal GISTs, in which recurrence was reported to occur in 35% [13]. Furthermore, four of eight patients with high-risk GIST died compared to none of the patients with low-risk tumors (0/5), indicating an impact of the Miettinen classification on long-term survival, even though it was not statistically significant (*P* = 0.0556) in our cohort due to the small number of cases. Our findings are in accordance with a previous study on duodenal GISTs [11], in which the authors identified the classification as high-risk GIST as the only predictor for disease recurrence [11]. In a study on 90 duodenal GISTs, however, only the mitotic rate predicted relapse in multivariate analysis [13]. Furthermore, in univariate analyses, age and ECOG performance state had an impact on overall survival, and necrosis, spindle cell morphology, tumor size, and mitotic rate were predictors for relapse [13]. In the present study, the ECOG state and necrosis were not assessed. Age, spindle cell differentiation, tumor size, and mitotic rate were not associated with a higher rate of recurrence.

Mutations of the *KIT* or *PDGFRA* gene have been identified as primary steps in tumorigenesis of GISTs [7]. In this study, ten tumors (76.9%) showed mutations of the *KIT* gene, and two tumors (15.4%) were “wild-type” GISTs. Four patients had an exon 9 mutation (30.8%) and six patients an exon 11 mutation (46.2%) [3, 18, 19]. *KIT* exon 9 mutations are reported to occur in 13–15%, exon 11 mutations in 66.1–70% (76% in duodenal GISTs), and wild-type GISTs making up approximately 10–15% [3, 13, 18, 20]. Thus, in the present study, *KIT* exon 9 mutations were observed slightly more frequent and exon 11 mutations less frequent than reported previously in the literature for GISTs. *KIT* exon 11 mutations are known to harbor a less favorable prognosis than *KIT* exon 9 mutations and are at high metastatic risk [20]. Also, point mutations are generally associated with a clinically more favorable course as compared to deletions [21]. However, exon 11 mutations show a better response to targeted treatment with imatinib mesylate than do exon 9 mutations [20].

Losses at 1p, 15q and 22q, as detected by CGH, are imbalances typical of small intestinal GISTs, being observed in 88%, 59%, and 82% of cases, respectively [22]. Especially, combined losses at 1p and 15q are described to occur in 75% of intestinal GISTs [22]. As reported previously, losses at 22q being present in 75% of our cases with tumor progress, are associated with an unfavorable cytogenetic sub-pathway and significantly more additional imbalances than tumors without −22q, reflecting an increased capacity for cytogenetic complexity [7]. GISTs with −22q are significantly more often high-risk tumors, behave clinically malignant, and have a poorer disease-free survival [7].

In a previous study, GISTs classified as probably benign or of low malignant potential had a smaller mean number of aberrations than those evaluated as probably malignant (4.6 versus 7.4) [22]. In our study, the mean number of 5.4 aberrations per tumor indicates that the tumors display a moderate degree of genetic instability and thus range between benign and malignant risk potential. Of all 13 tumors, 6 displayed ≥5 aberrations, thus tending towards a rather instable karyotype with genetic progression. CGH can be used as helpful additional method to assess the risk of malignancy or progression in duodenal GISTs and might help in deciding for or against (neo-) adjuvant treatment.

Concerning the operative procedure, the authors of previous studies preferred segmental duodenectomy to duodenopancreatectomy since this procedure has a lower operative morbidity while providing comparable oncological results [5, 6, 9, 11–13, 23]. However, in one of these studies, only tumors with <5 mitoses/50 HPFs, a mean size of 3.5 cm, and very low/low/intermediate risk were included and treated with limited surgery [12]. In smaller tumors, measuring ≤5 cm in size, limited resection seems to be favorable. Duodenal GISTs of >5 cm are already classified as high-risk tumors according to the Miettinen classification, irrespective of the mitotic count. In these cases and in locally advanced tumors neoadjuvant treatment is an option to reduce tumor size [13]. Adjuvant therapy should follow. However, in our study, four high-risk patients did not receive adjuvant therapy—as at the time, adjuvant therapy (i.e., therapy within the first weeks after operation) was not generally performed after complete resection of the tumor. To our knowledge, two of these four patients died of tumor progression. In our study, one of the patients, who underwent segmental duodenectomy, died within 30 days after the operation in contrast to none of the patients with duodenopancreatectomy, but no significant advantage of one or the other operative method was detectable.

In conclusion, complete surgical resection is the only curative treatment for duodenal GISTs. Since both, limited and extended surgery yield comparable survival rates, tumor size and location in regard to the papilla of Vater [11], associated diseases and the patient's performing state should be considered when deciding between segmental duodenectomy and pylorus-preserving duodenopancreatectomy. If duodenopancreatectomy is necessary, it has no impact on overall survival and recurrence rates in experiences centers. Neoadjuvant imatinib treatment might be an option and is tested in clinical studies. The risk stratification according to the Miettinen criteria and the assessment of genomic aberrations by CGH are helpful in predicting the biological behavior and clinical course of duodenal GISTs.
